# Sufficient-dose Brentuximab vedotin improves prognosis in patients with classic Hodgkin lymphoma: a single-center real-world study in China

**DOI:** 10.3389/fonc.2026.1889698

**Published:** 2026-07-06

**Authors:** Zhangyuting He, Huiying Zhu, Chong Wei, Danqing Zhao, Jing Ruan, Wei Zhang, Daobin Zhou, Yan Zhang

**Affiliations:** 1 Department of Hematology, Peking Union Medical College Hospital, Chinese Academy of Medical Sciences and Peking Union Medical College, Beijing, China; 2 Department of Internal Medicine, Peking Union Medical College Hospital, Chinese Academy of Medical Sciences and Peking Union Medical College, Beijing, China

**Keywords:** Brentuximab vedotin, classic Hodgkin lymphoma, dose intensity, real-word analysis, real-world evidence

## Abstract

**Background:**

Classic Hodgkin lymphoma (cHL) is highly curable for most patients with cytotoxic chemotherapy, while targeted agents such as brentuximab vedotin (BV) have been incorporated into frontline regimens. This single-center retrospective study aimed to compare the efficacy and safety of a local ABVD-like regimen and a BV-containing A-AVD-like regimen in Chinese patients with cHL.

**Methods:**

A total of 243 cHL patients were enrolled, including 192 in ABVD and 51 in A-AVD. Baseline demographics, treatment outcomes, survival, and adverse events were analyzed; the median follow-up was 21.47 months. Subgroup analysis was performed including Ann Arbor stage and dose of Brentuximab Vedotin (BV).

**Results:**

At baseline, the A-AVD group had a significantly higher proportion of elderly patients (≥60 years) compared to the ABVD group (25.5% vs. 9.4%, *p* = 0.002). First-evaluation complete response (CR: 69.3% vs. 72.5%, *p* = 0.549) and overall response rate (ORR: 93.8% vs. 98.0%, *p* = 0.312) were similar between ABVD and A-AVD. Overall progression-free survival (PFS) and overall survival (OS) showed no significant differences between the two regimens (PFS p=0.489; OS *p* = 0.230). The 2-year PFS rates were 71.1% for ABVD and 76.7% for A-AVD. However, subgroup analysis revealed that in patients with Ann Arbor stage III-IV, a sufficient dose of BV (≥1.15 mg/kg) significantly improved PFS compared to a reduced dose (<1.15 mg/kg) (2-year PFS 91.7% vs. 58.7%; p=0.036). Regarding safety, the A-AVD group exhibited a higher rate of peripheral neuropathy (47.1% vs. 5.2%, *p* < 0.001), while pulmonary toxicity events were numerically more frequent in the ABVD group. The incidences of grade 3–4 neutropenia were comparable between the two groups (17.7% in ABVD vs. 19.6% in A-AVD, *p* = 0.751).

**Conclusion:**

This is the first real-world study of frontline A-AVD-like therapy for cHL in China. Despite a higher proportion of elderly patients in A-AVD, the regimen still achieved a 2-year PFS rate of 76.7% and showed overall efficacy and survival outcomes comparable to the ABVD-like regimen. Notably, maintaining sufficient BV dose intensity (≥1.15 mg/kg) was significantly associated with superior PFS in patients with advanced-stage (Ann Arbor III-IV) disease. While A-AVD was associated with higher rates of peripheral neuropathy, ABVD showed a trend toward a higher risk of pulmonary toxicity. These findings support careful maintenance of BV dose intensity when clinically feasible, together with proactive toxicity management. Longer follow-up and larger prospective studies are needed to validate survival outcomes and toxicity profiles in Chinese patients.

## Introduction

Classic Hodgkin lymphoma (cHL) is a distinct subtype of lymphoid malignancy, accounting for approximately 10%–15% of all lymphoma cases (1). cHL is widely regarded as a paradigm of a highly curable malignancy, and most patients achieve favorable long-term outcomes with established cytotoxic regimens. The ABVD regimen (doxorubicin, bleomycin, vinblastine, dacarbazine) has long been the cornerstone of first-line treatment for advanced cHL. In early-stage disease, ABVD regimen could achieve 5-year progression-free survival (PFS) rates of 85-95% and overall survival (OS) exceeding 90% (2). In advanced-stage patients, ABVD regimen could achieve a 5-year OS of 75-85% (3). Nevertheless, a clinically important minority of patients, particularly those with resistant or refractory disease, still fare poorly. Patients with an International Prognostic Score (IPS) ≥4, bulky disease, or other high-risk features have lower complete response (CR) rates and higher recurrence risk when treated with ABVD alone. In addition, bleomycin is associated with dose-dependent pulmonary toxicity, which can cause complications such as interstitial pneumonitis and pulmonary fibrosiss (2).

Pathologically, cHL is defined by the presence of Reed-Sternberg cells, which are typically CD30-positive (>90%) (4–6). The unique tumor microenvironment of cHL, rich in inflammatory cells and immunosuppressive cytokines, has driven the development of targeted therapies against CD30 and immune checkpoint pathways (7). Brentuximab vedotin (BV), an anti-CD30 antibody-drug conjugate, exerts its therapeutic effect through targeted delivery of monomethyl auristatin E to CD30-positive cells, inducing apoptosis and disrupting microtubule formation (4, 8, 9). Rather than replacing cytotoxic chemotherapy, BV has been incorporated into chemotherapy backbones to improve disease control while avoiding bleomycin-related pulmonary toxicity. In advanced-stage disease, A-AVD showed a significantly higher 2-year PFS rate than ABVD in ECHELON-1 and subsequent analyses (10–14). Real-world evidence further supports the clinical activity of A-AVD, also emphasizing the need to balance efficacy, peripheral neuropathy, and dose modifications (15).

In China, BV was approved for marketing in 2020 and included in the national medical insurance program in 2023. Its application scope has gradually expanded, and it has been incorporated into clinical guidelines. There was a lack of a summary of real-world application experience in China. Previous studies have shown that Chinese patients with hematological malignancies may have a higher incidence of certain toxicities when using BV, which may lead to more frequent dose adjustments and potentially compromise efficacy (8, 16).

This study aimed to compare the efficacy and safety of local ABVD-like and A-AVD-like regimens in Chinese patients with cHL through a single-center retrospective real-world analysis. The specific research objective was to compare response, PFS, OS, and adverse events between the two regimens in a heterogeneous real-world cohort.

## Methods

### Population and data collection

This single center, retrospective study was conducted in accordance with the ethical principles of the 1964 Helsinki Declaration and approved by the Ethics Committee of Peking Union Medical College Hospital (approval number: Y4682). All patient data were anonymized and identified prior to analysis. Between November 2007 and August 2024, adult patients (≥18 years) with untreated cHL according to National Comprehensive Cancer Network (NCCN) criteria who received ABVD or A-AVD chemotherapy at Peking Union Medical College Hospital (PUMCH) were included. Inclusion criteria were: pathologically confirmed cHL by hematopathology review; measurable disease under Lugano classification; complete baseline and follow-up data; and at least one time efficacy assessment. Exclusion criteria include the occurrence of pathological changes during the disease course; the treatment regimen involves other targeted or chemotherapeutic agents. Clinical data were retrospectively extracted from electronic medical records, including demographics, disease characteristics (Ann Arbor stage, extranodal involvement), treatment details, and follow-up outcomes. NCCN criteria for early unfavorable stage disease included the following risk factors: erythrocyte sedimentation rate > 50mm/h, presence of B symptoms, involvement of more than 3 nodal sites, a mediastinal mass ratio of 1:3 (maximum width of mass/maximum intrathoracic diameter), or a mass larger than 10 cm in any dimension. Patients with advanced stage were stratified into low-IPS-risk (score 0–3) and high-IPS-risk (score 4–7) groups. And pathological subtypes were confirmed.

### Therapeutic approaches

In this retrospective real-world cohort, the ABVD and A-AVD labels refer to the institutional ABVD-like and A-AVD-like regimens used at our center. Epirubicin and vinorelbine were used as local substitutions for doxorubicin and vinblastine, respectively, within the same anthracycline and vinca alkaloid drug classes. This substitution reflected long-standing local practice, drug availability and reimbursement, and physician discretion during the retrospective enrollment period; the exact components and doses are therefore specified here. The ABVD-like regimen contained Epirubicin (40 mg/m², IV, day 1 and 15), Bleomycin (10 mg/m², IV, day 1 and 15), Vinorelbine (4 mg, IV, day 1 and 15), Dacarbazine (25 mg/m², IV, day 1 and 15) every 28 days for 6 cycles. The A-AVD-like regimen contains BV (1.2 mg/kg, IV, day 1 and 15), Epirubicin (40 mg/m², IV, day 1 and 15), Vinorelbine (4 mg, IV, day 1 and 15), Dacarbazine (25 mg/m², IV, day 1 and 15) every 28 days for 6 cycles. The decision to administer treatment was based on clinical indications and patient-specific characteristics. Dose modifications were made according to toxicities at the physician’s discretion. After completion of frontline chemotherapy, sequential radiotherapy or ASCT consolidation was not routinely administered after successful treatment; it was selectively considered according to physician assessment of disease risk, response, residual disease, and patient fitness.

### Efficacy and safety analysis

Response evaluations were performed using positron emission tomography/computed tomography (PET-CT) based on the 2014 Lugano classification using the 5-point scale, including CR (no evidence of disease), partial response (PR, ≥50% reduction in tumor size), stable disease (SD), or progressive disease (PD). A Deauville score of 1–3 on a PET/CT was considered as CR, whereas a Deauville score of 4 and 5 represented PR. Interim assessments were conducted after cycles 2 or 4, with final end-of-treatment (EOT) evaluation at cycles 6. PFS is the time from diagnosis to disease progression, death from any cause, or last follow-up. OS is defined as the time from diagnosis to death from any cause or the last follow-up. Adverse events (AEs) were recorded according to the National Cancer Institute Common Terminology Criteria (CTCAE, version 5.0).

### Statistical methods

Baseline characteristics were compared between ABVD group and A-AVD group with the Kruskal–Wallis H test, χ2 tests, or Fisher exact test. All statistical comparisons adhered to a two-sided approach, with statistical significance set at a p-value < 0.05. PFS, and OS were summarized using the Kaplan–Meier method. Survival endpoints were analyzed with a Cox proportional hazards model. Exploratory subgroup Cox analyses were performed to compare PFS between A-AVD and ABVD across key baseline strata, and the results were summarized in a forest plot. A sensitivity analysis excluding patients who received sequential radiotherapy or ASCT consolidation was also performed to assess the potential influence of post-chemotherapy consolidation on regimen comparisons. The above analyses were conducted using SPSS, version 27.0.

## Results

### Baseline demographics and clinical characteristics

A total of 243 patients with cHL were enrolled in this study, including 192 in the ABVD group and 51 in the A-AVD group. The median age for the entire cohort was 31 years. While the median age did not differ significantly between groups (31 vs. 33 years, *p* = 0.323), the A-AVD group had a significantly higher proportion of elderly patients (≥60 years) compared to the ABVD group (25.5% vs. 9.4%, *p* = 0.002). The distribution of gender was comparable between the two arms (male proportion: 54.2% in ABVD vs. 45.1% in A-AVD, *p* = 0.249).

Regarding pathological subtypes, nodular sclerosis was the most common (53.5%), followed by mixed cellularity (19.8%) and lymphocyte-rich (14.0%), with no significant statistical differences between groups (*p* = 0.567). In terms of disease staging, 44.9% of patients had early-stage disease (Ann Arbor stage I-II), and 55.1% had advanced-stage disease (III-IV). The proportion of stage IV patients was numerically higher in the A-AVD group compared to the ABVD group (49.0% vs. 34.9%, *p* = 0.067). Among patients with advanced disease (III-IV), 64.9% were categorized as low-to-intermediate risk (IPS 0–3) and 35.1% as high-risk (IPS 4–7), showing a balanced distribution between the two groups (*p* = 0.798).

Other clinical characteristics, including B symptoms (47.3%), bulky disease (36.6%), and involvement of the lungs (18.9%), bones (24.7%), spleen (18.5%), and liver (7.8%), showed no significant statistical differences between the two regimens. In the overall cohort, 14.0% of patients received sequential radiotherapy consolidation (16.1% in the ABVD group vs. 5.9% in the A-AVD group, *p* = 0.060), and 4.5% received ASCT consolidation (5.7% vs. 0.0%, *p* = 0.127). Detailed baseline characteristics are summarized in **Table 1**.

**Table 1:** Baseline characteristics of all patients stratified by treatment.

Characteristic	Overall N = 243	ABVD N = 192	A-AVD N = 51	p-value
Age, years, Median (Q1, Q3)	31 (24, 49)	31 (24, 47)	33 (23, 60)	0.3231
Age, years, n (%)				0.0022
< 60	212 (87.2%)	174 (90.6%)	38 (74.5%)	
≥ 60	31 (12.8%)	18 (9.4%)	13 (25.5%)	
Gender, n (%)				0.2492
Female	116 (47.7%)	88 (45.8%)	28 (54.9%)	
Male	127 (52.3%)	104 (54.2%)	23 (45.1%)	
Pathology, n (%)				0.5673
Mixed Cellularity	48 (19.8%)	39 (20.3%)	9 (17.6%)	
Nodular Sclerosis	130 (53.5%)	102 (53.1%)	28 (54.9%)	
Lymphocyte-Rich	34 (14.0%)	29 (15.1%)	5 (9.8%)	
Lymphocyte-Depleted	2 (0.8%)	2 (1.0%)	0 (0.0%)	
Unclassifiable	29 (11.9%)	20 (10.4%)	9 (17.6%)	
Ann Arbor, n (%)				0.0673
I	8 (3.3%)	8 (4.2%)	0 (0.0%)	
II	101 (41.6%)	86 (44.8%)	15 (29.4%)	
III	42 (17.3%)	31 (16.1%)	11 (21.6%)	
IV	92 (37.9%)	67 (34.9%)	25 (49.0%)	
B symptoms, n (%)	115 (47.3%)	89 (46.4%)	26 (51.0%)	0.5562
IPS, Median (Q1, Q3)	3.00 (2.00, 4.00)	3.00 (2.00, 4.00)	2.50 (1.00, 4.00)	0.5661
Ann Arbor III-IV, IPS 0-3	87 (64.9%)	63 (64.3%)	24 (66.7%)	0.7982
Ann Arbor III-IV, IPS 4-7	47 (35.1%)	35 (35.7%)	12 (33.3%)	
Bulky Disease, n (%)	89 (36.6%)	66 (34.4%)	23 (45.1%)	0.1582
Lung Involvement, n (%)	46 (18.9%)	35 (18.2%)	11 (21.6%)	0.5882
Bone Involvement, n (%)	60 (24.7%)	44 (22.9%)	16 (31.4%)	0.2132
Spleen Involvement, n (%)	45 (18.5%)	31 (16.1%)	14 (27.5%)	0.0652
Liver Involvement, n (%)	19 (7.8%)	16 (8.3%)	3 (5.9%)	0.7713
Sequential RT Consolidation, n (%)	34 (14.0%)	31 (16.1%)	3 (5.9%)	0.0602
ASCT Consolidation, n (%)	11 (4.5%)	11 (5.7%)	0 (0.0%)	0.1273

ABVD, Epirubicin, Bleomycin, Vinorelbine, and Dacarbazine; A-AVD, Brentuximab vedotin, Epirubicin, Vinorelbine, and Dacarbazine; IPS, International Prognostic Score; RT, Radiotherapy; ASCT, Autologous Stem Cell Transplantation; Q1, First Quartile; Q3, Third Quartile.

For the 36 patients with advanced-stage disease in the A-AVD cohort, we further compared the baseline characteristics of the reduced-dose (<1.15 mg/kg, n = 22) and sufficient-dose (≥1.15 mg/kg, n = 14) subgroups. As detailed in **Supplementary Table 1**, all demographic and clinical features were well balanced between the two dosage arms.

### Efficacy evaluation

The median overall follow-up duration of this study was 21.47 months, with 22.57 months in the ABVD group and 17.60 months in the A-AVD group. Efficacy results for the overall cohort are presented in **Supplementary Table 2**, while the subgroup efficacy based on Ann Arbor staging is detailed in **Supplementary Table 3** (stage I-II) and **Supplementary Table 4** (stage III-IV). For the first (interim) evaluations, the two groups achieved comparable CR (69.3% in ABVD vs. 72.5% in A-AVD, *p* = 0.549) and ORR (93.8% vs. 98.0%, *p* = 0.312). For the end-of-treatment evaluations, the CR/CMR remained robust in both groups (72.2% vs. 78.3%, *p* = 0.809), with an ORR of 82.4% in the ABVD group and 84.8% in the A-AVD group (*p* = 0.863) excluding patients with missing assessments.

Subgroup analysis based on Ann Arbor staging revealed high response rates in patients with stage I-II disease (**Supplementary Table 3**), with an interim ORR of 96.8% in the ABVD group and 100.0% in the A-AVD group (p>0.999). For patients with advanced-stage disease (III-IV, **Supplementary Table 4**), the interim ORR remained high at 90.8% and 97.2%, respectively (p=0.287). The end-of-treatment ORR rates for the advanced-stage subgroup were consistent across both regimens (83.0% vs. 81.3%, *p* = 0.840).

### Survival analysis

**Figure 1:**
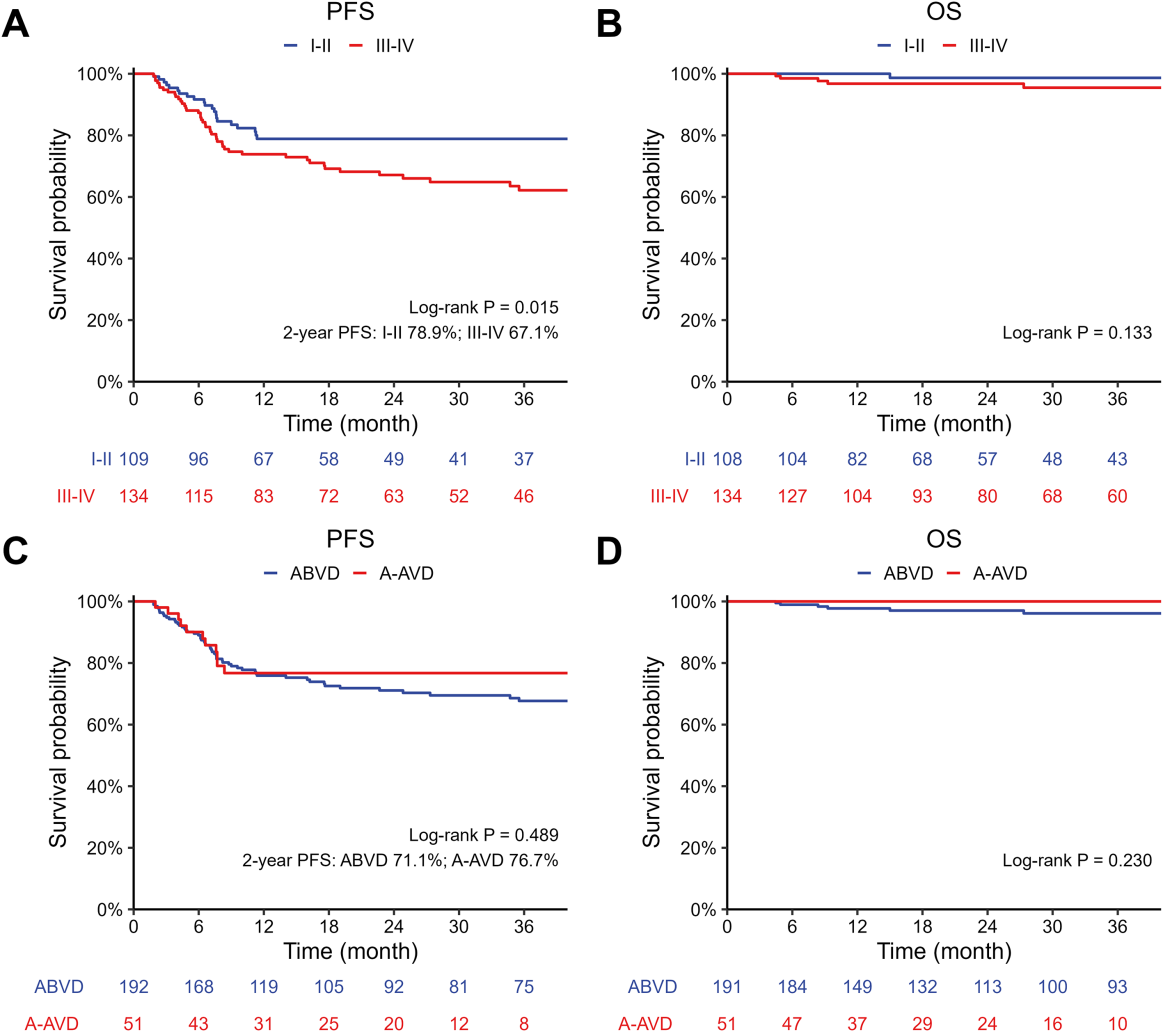
Kaplan-Meier estimates of survival outcomes. **(A)** Progression-free survival (PFS) stratified by early (Ann Arbor I-II) and advanced (Ann Arbor III-IV) disease stages. **(B)** Overall survival (OS) stratified by early and advanced disease stages. **(C)** PFS comparing the ABVD and A-AVD regimens in the overall cohort. **(D)** OS comparing the ABVD and A-AVD regimens in the overall cohort.

Survival analysis was performed using the Kaplan-Meier method. As expected, patients with early-stage disease (Ann Arbor I-II) demonstrated significantly superior PFS (2-year PFS 78.9% vs. 67.1%; log-rank p = 0.015, **Figure 1A**) and a trend toward better OS (Log-rank *p* = 0.133, **Figure 1B**) compared to those with advanced-stage disease (Ann Arbor III-IV). When comparing treatment regimens across the entire cohort, PFS showed no significant difference between the ABVD and A-AVD groups (2-year PFS 71.1% vs. 76.7%; log-rank p = 0.489, **Figure 1C**. Similarly, OS did not differ significantly between the two treatment arms (Log-rank *p* = 0.230, **Figure 1D**).

### Prognostic factors

Univariate Cox regression analysis (**Supplementary Table 5**) identified that female gender (HR = 0.57, 95% CI: 0.35-0.92, *p* = 0.023) and nodular sclerosis pathology (HR = 0.56, 95% CI: 0.33-0.97, *p* = 0.037) were associated with a significantly better PFS. Conversely, liver involvement (HR = 2.28, 95% CI: 1.20-4.33, *p* = 0.012) was a significant adverse prognostic factors. Factors such as age, B symptoms, bulky disease, and other organ involvements did not show statistical significance in the univariate analysis.

### Subgroup analysis

**Figure 2:**
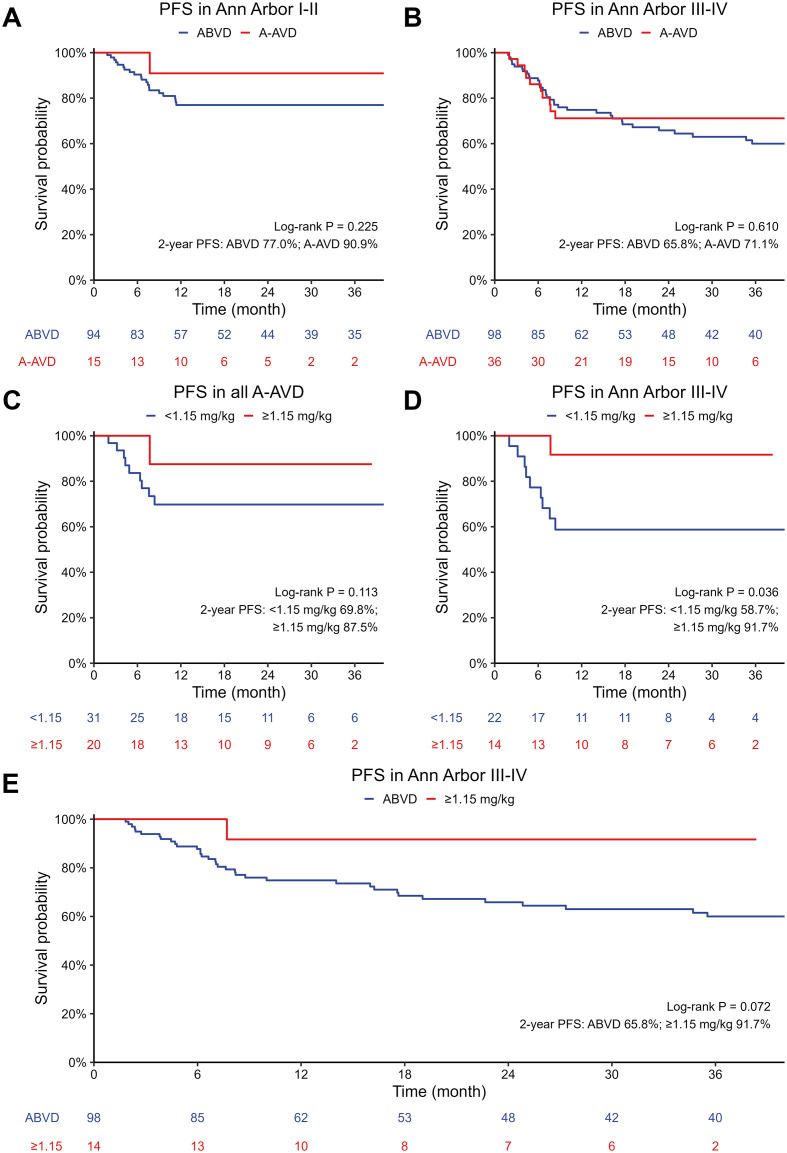
Kaplan-Meier estimates of progression-free survival (PFS) subgroup analyses. **(A)** PFS comparing ABVD and A-AVD regimens in early-stage (Ann Arbor I-II) disease. **(B)** PFS comparing ABVD and A-AVD regimens in advanced-stage (Ann Arbor III-IV) disease. **(C)** PFS stratified by BV dose (<1.15 mg/kg vs. ≥1.15 mg/kg) in the overall A-AVD cohort. **(D)** PFS stratified by BV dose in the subgroup of A-AVD patients with advanced-stage disease. **(E)** PFS comparing advanced-stage patients treated with ABVD versus those receiving sufficient-dose A-AVD (≥1.15 mg/kg).

Further subgroup survival analysis revealed that PFS did not differ significantly between the ABVD and A-AVD regimens when stratified by early-stage (Ann Arbor I-II; 2-year PFS 77.0% vs. 90.9%; log-rank p = 0.225; **Figure 2A**) or advanced-stage (Ann Arbor III-IV; 2-year PFS 65.8% vs. 71.1%; log-rank p = 0.610; **Figure 2B**) disease. In the overall A-AVD cohort, patients receiving a sufficient BV dose (≥1.15 mg/kg) showed a trend toward better PFS than those receiving a reduced dose (<1.15 mg/kg), though it did not reach statistical significance (2-year PFS 87.5% vs. 69.8%; p = 0.113; **Figure 2C**). In patients with advanced disease (Ann Arbor III-IV), receiving a sufficient dose of BV (≥1.15 mg/kg) resulted in significantly superior PFS compared with the reduced-dose group (2-year PFS 91.7% vs. 58.7%; p = 0.036; **Figure 2D**). Furthermore, among advanced-stage patients, the sufficient-dose A-AVD group showed a strong trend toward improved PFS compared with the ABVD cohort (2-year PFS 91.7% vs. 65.8%; log-rank p = 0.072; **Figure 2E**).

To address potential confounding by post-chemotherapy consolidation, we performed a sensitivity analysis excluding patients who received sequential radiotherapy or ASCT consolidation. Among patients without consolidation (ABVD n=151; A-AVD n=48), regimen comparison remained non-significant (2-year PFS 67.5% vs. 75.1%; log-rank p=0.361). In advanced-stage patients without consolidation (ABVD n=74; A-AVD n=36), A-AVD showed numerically higher 2-year PFS but the difference was not statistically significant (60.3% vs. 71.1%; log-rank p=0.298).

**Figure 3:**
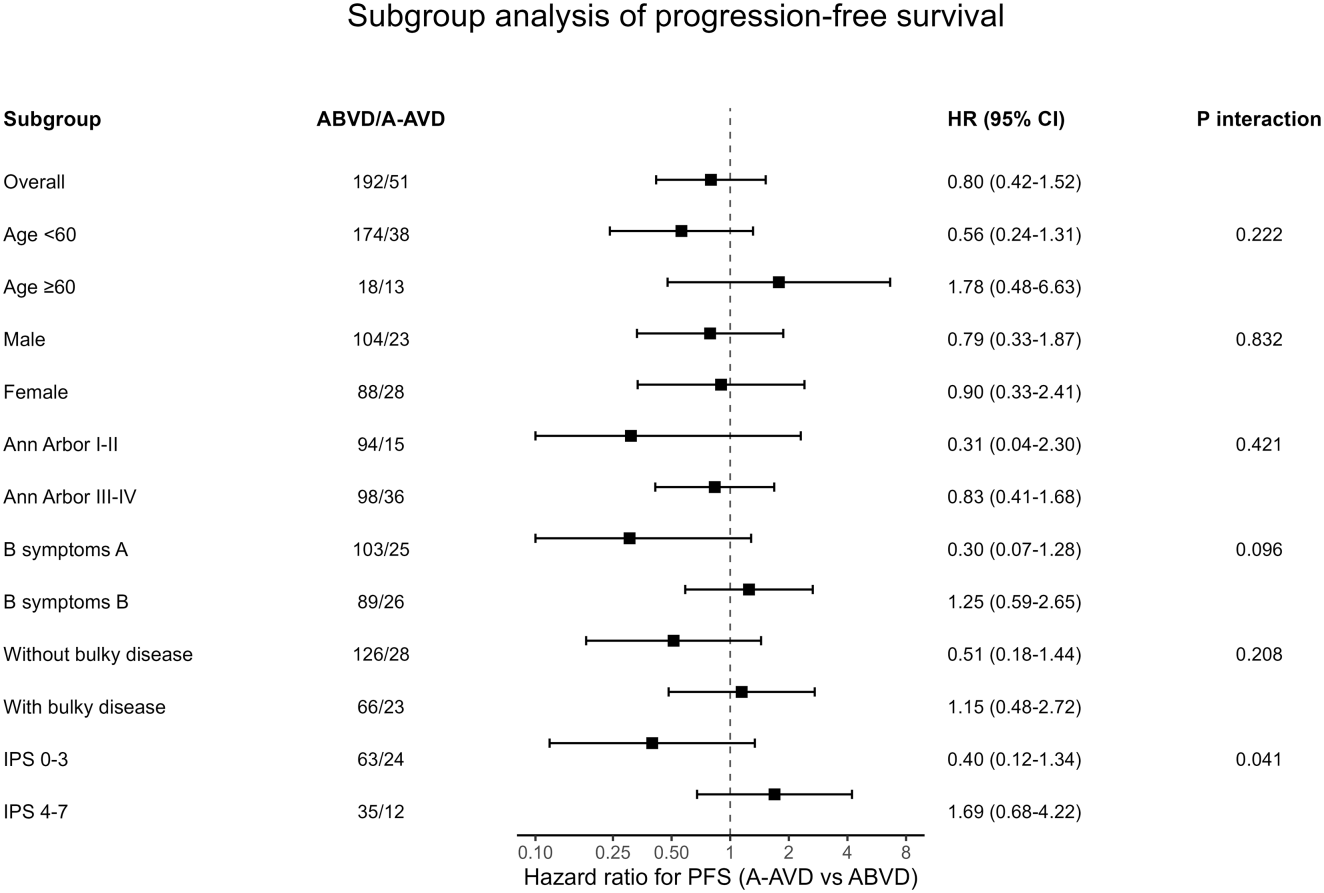
Exploratory subgroup Cox analysis of progression-free survival comparing A-AVD with ABVD across key baseline strata. Hazard ratios (HRs) are shown for A-AVD versus ABVD; HR <1 favors A-AVD. CI, confidence interval; IPS, International Prognostic Score.

An exploratory Cox forest plot comparing A-AVD with ABVD across key baseline subgroups is presented in **Figure 3**. Overall, the HR for PFS with A-AVD versus ABVD was 0.80 (95% CI: 0.42-1.52). HR estimates favored A-AVD in several subgroups, but most confidence intervals crossed 1.0, and interaction tests were generally non-significant; the IPS subgroup interaction should be interpreted cautiously because of the small subgroup size and limited number of events.

### Safety analysis

AEs were collated and summarized based on medical records, as shown in **Supplementary Table 6**. The incidence of all AEs was comparable between the ABVD and A-AVD groups (56.2% vs. 60.8%, *p* = 0.551). Furthermore, there was no statistically significant difference in the occurrence of Grade 3–4 AEs overall between the two arms (21.9% vs. 33.3%, *p* = 0.093). Specifically, the incidence of Grade 3–4 neutropenia showed no significant difference between the two groups (17.7% vs. 19.6%, *p* = 0.751). The occurrences of other specific AEs, including pulmonary toxicity and anemia, were comparable across both cohorts. However, the A-AVD group exhibited significantly higher rates of peripheral neuropathy (47.1% vs. 5.2%, *p* < 0.001), nausea (31.4% vs. 12.0%, *p* = 0.001), and rash (21.6% vs. 6.2%, *p* = 0.001), as well as a higher incidence of Grade 3–4 thrombocytopenia (11.8% vs. 3.6%, *p* = 0.041).

## Discussion

This study is the first real-world study in China evaluating the frontline use of a BV-containing A-AVD-like regimen in a broad cohort of patients with cHL. Our findings provide two clinically relevant observations for optimizing frontline therapy in routine practice. First, despite a significantly higher proportion of elderly patients (≥60 years) in the A-AVD group, the regimen demonstrated comparable overall efficacy and a 2-year PFS of 76.7%, suggesting that advanced age alone should not preclude consideration of BV-containing therapy in carefully selected patients. Second, maintaining sufficient BV dose intensity (≥1.15 mg/kg) was significantly associated with superior PFS in patients with advanced-stage (Ann Arbor III-IV) disease. This highlights the delicate balance required between toxicity management and treatment efficacy.

Regarding safety, the A-AVD group exhibited a higher incidence of peripheral neuropathy, while the ABVD group showed a numerically higher risk of pulmonary toxicity. Studies have reported that in cHL patients receiving A-AVD, the incidence of peripheral neuropathy is 67%-80% (Grade 3 accounts for 10%-12%). Most patients’ symptoms can alleviate in approximately 16 weeks, and the doses of BV or vinblastine can be adjusted according to the severity (17). This is consistent with the international safety profile of the A-AVD regimen. However, due to the potential incomplete recording of adverse events in medical records, the overall medication side effects may be underestimated.

Compared with ECHELON-1 and other prospective studies, the present retrospective study showed a directionally similar but statistically non-significant trend favoring the A-AVD regimen (10, 18–20). Several factors may explain the absence of a significant overall PFS advantage. First, because A-AVD was introduced later than ABVD in China, the median follow-up time of the ABVD group (22.57 months) was longer than that of the A-AVD group (17.60 months). Longer follow-up may clarify whether a survival separation emerges. Second, patients in clinical trials are strictly selected, whereas this real-world cohort included older and more heterogeneous patients. For elderly cHL patients, fitness-guided treatment remains essential because chemotherapy can increase pulmonary toxicity and myelosuppression (21, 22).

Real-world implementation of consolidation therapies, such as radiotherapy and ASCT, can confound regimen comparisons because these interventions are applied selectively according to risk, response, residual disease, and physician judgment. In our cohort, sequential RT and ASCT consolidation were more frequently used in the ABVD group. After excluding patients who received sequential RT or ASCT consolidation, the PFS comparison between ABVD and A-AVD remained non-significant in the overall cohort and in the advanced-stage cohort, supporting the robustness of the main finding while also underscoring the inherent confounding of retrospective real-world analyses (4).

Finally, BV dose reduction in the A-AVD group may be an important contributor to the observed efficacy pattern (23, 24). While overall PFS did not significantly differ between the <1.15 mg/kg and ≥1.15 mg/kg groups, patients with Ann Arbor stage III-IV disease derived a significant PFS benefit from sufficient-dose BV (≥1.15 mg/kg) (p=0.036). This association suggests that dose reduction of BV, often instituted because of toxicities such as peripheral neuropathy, may reduce antineoplastic efficacy in high-risk advanced patients (17). Clinicians should therefore manage adverse events proactively and individualize dose modifications rather than reducing BV prematurely when toxicity is manageable. Both BV and vinca alkaloids are microtubule-targeting agents and may increase chemotherapy-induced peripheral neuropathy; graded adjustment strategies may help balance adverse effects and therapeutic efficacy (1, 23).

Several limitations must be acknowledged. First, the unequal group sizes and shorter follow-up for the A-AVD group may have reduced statistical power. Second, the retrospective single-center design limits causal inference. Third, the ABVD and A-AVD labels in this study refer to local ABVD-like and A-AVD-like regimens using epirubicin and vinorelbine rather than doxorubicin and vinblastine; this should be considered when comparing our results with trials using the original ABVD or A-AVD components. Fourth, sequential RT or ASCT consolidation was applied according to real-world physician assessment and may have introduced confounding, although sensitivity analyses excluding these patients showed similar overall conclusions. Finally, subgroup and forest-plot analyses were exploratory and should be interpreted cautiously because of limited events in some strata.

As the first real-world study of frontline A-AVD-like therapy for cHL in China, this study showed that despite the higher proportion of elderly patients in the A-AVD group, the regimen still achieved overall efficacy and survival outcomes comparable to those of the ABVD-like group. No significant overall efficacy advantage was observed, likely because of differences in follow-up time, real-world consolidation practices, and BV dose reduction. Maintaining sufficient BV dose intensity was associated with improved PFS in advanced-stage patients, highlighting the need to balance toxicity management and efficacy maintenance in clinical practice. Future studies with larger sample sizes and longer follow-up are needed to validate the long-term benefits of BV-based regimens in Chinese patients.

## Data Availability

The original contributions presented in the study are included in the article/**Supplementary Material**. Further inquiries can be directed to the corresponding author.
