# Supplementary material for: Sufficient-dose Brentuximab vedotin improves prognosis in patients with classic Hodgkin lymphoma: a single-center real-world study in China
Source: Front Oncol. 2026 Jul 6;16:1889698. doi: 10.3389/fonc.2026.1889698 (PMC13381218; doi:10.3389/fonc.2026.1889698)
Supplement: Supplementary file 1 [file Table1.docx]

Supplementary Table S1. Baseline characteristics of advanced patients stratified by BV dose.

| Characteristic | Overall  N = 36 | ˂1.15mg/kg  N = 22 | ≥1.15mg/kg  N = 14 | p-value |
| --- | --- | --- | --- | --- |
| Age, years, Median (Q1, Q3) | 33 (28, 64) | 33 (23, 64) | 34 (31, 54) | 0.329^1^ |
| Age, years, n (%) |  |  |  | 0.467^2^ |
| ˂ 60 | 25 (69.4%) | 14 (63.6%) | 11 (78.6%) |  |
| ≥ 60 | 11 (30.6%) | 8 (36.4%) | 3 (21.4%) |  |
| Gender, n (%) |  |  |  | 0.878^3^ |
| Female | 16 (44.4%) | 10 (45.5%) | 6 (42.9%) |  |
| Male | 20 (55.6%) | 12 (54.5%) | 8 (57.1%) |  |
| Pathology, n (%) |  |  |  | 0.113^2^ |
| Mixed Cellularity | 8 (22.2%) | 7 (31.8%) | 1 (7.1%) |  |
| Nodular Sclerosis | 20 (55.6%) | 12 (54.5%) | 8 (57.1%) |  |
| Lymphocyte-Rich | 5 (13.9%) | 1 (4.5%) | 4 (28.6%) |  |
| Lymphocyte-Depleted | 3 (8.3%) | 2 (9.1%) | 1 (7.1%) |  |
| Unclassifiable | 0 (0.0%) | 0 (0.0%) | 0 (0.0%) |  |
| Ann Arbor, n (%) |  |  |  | 0.716^2^ |
| III | 25 (69.4%) | 16 (72.7%) | 9 (64.3%) |  |
| IV | 11 (30.6%) | 6 (27.3%) | 5 (35.7%) |  |
| B symptoms, n (%) | 23 (63.9%) | 16 (72.7%) | 7 (50.0%) | 0.166^3^ |
| IPS, n (%) |  |  |  | 0.292^2^ |
| Ann Arbor III-IV，IPS 0-3 | 24 (66.7%) | 13 (59.1%) | 11 (78.6%) |  |
| Ann Arbor III-IV，IPS 4-7 | 12 (33.3%) | 9 (40.9%) | 3 (21.4%) |  |
| Bulky Disease, n (%) | 15 (41.7%) | 10 (45.5%) | 5 (35.7%) | 0.563^3^ |
| Lung Involvement, n (%) | 10 (27.8%) | 6 (27.3%) | 4 (28.6%) | >0.999^2^ |
| Bone Involvement, n (%) | 15 (41.7%) | 9 (40.9%) | 6 (42.9%) | 0.908^3^ |
| Spleen Involvement, n (%) | 13 (36.1%) | 7 (31.8%) | 6 (42.9%) | 0.501^3^ |
| Liver Involvement, n (%) | 3 (8.3%) | 2 (9.1%) | 1 (7.1%) | >0.999^2^ |
| Sequential RT Consolidation, n (%) | 0 (0.0%) | 0 (0.0%) | 0 (0.0%) | >0.999^2^ |
| ASCT Consolidation, n (%) | 0 (0.0%) | 0 (0.0%) | 0 (0.0%) | >0.999^2^ |
| ^1^Wilcoxon rank sum test | | | | |
| ^2^Fisher's exact test  ^3^Pearson's Chi-squared test | | | | |

Abbreviations: ABVD, Epirubicin, Bleomycin, Vinorelbine, and Dacarbazine; A-AVD, Brentuximab vedotin, Epirubicin, Vinorelbine, and Dacarbazine; BV = Brentuximab vedotin; IPS = International Prognostic Score; RT = Radiotherapy; ASCT = Autologous Stem Cell Transplantation; Q1 = First Quartile; Q3 = Third Quartile.

Supplementary Table S2. Evaluation of interim and end of treatment

| Characteristic | Overall  N = 243 | ABVD  N = 192 | A-AVD  N = 51 | p-value |
| --- | --- | --- | --- | --- |
| First (interim) evaluation, n (%) |  |  |  | 0.549^1^ |
| CR | 170 (70.0%) | 133 (69.3%) | 37 (72.5%) |  |
| PR | 60 (24.7%) | 47 (24.5%) | 13 (25.5%) |  |
| PD | 13 (5.3%) | 12 (6.3%) | 1 (2.0%) |  |
| ORR | 230 (94.7%) | 180 (93.8%) | 50 (98.0%) | 0.312^1^ |
| Deauville Score, n (%) |  |  |  | 0.333^1^ |
| 1 | 51 (21.3%) | 39 (20.7%) | 12 (23.5%) |  |
| 2 | 78 (32.6%) | 67 (35.6%) | 11 (21.6%) |  |
| 3 | 51 (21.3%) | 38 (20.2%) | 13 (25.5%) |  |
| 4 | 47 (19.7%) | 34 (18.1%) | 13 (25.5%) |  |
| 5 | 12 (5.0%) | 10 (5.3%) | 2 (3.9%) |  |
| End evaluation, n (%) |  |  |  | 0.809^1^ |
| CR/CMR | 171 (73.4%) | 135 (72.2%) | 36 (78.3%) |  |
| PR/PMR | 22 (9.4%) | 19 (10.2%) | 3 (6.5%) |  |
| SD | 1 (0.4%) | 1 (0.5%) | 0 (0.0%) |  |
| PD | 39 (16.7%) | 32 (17.1%) | 7 (15.2%) |  |
| ORR | 193 (82.8%) | 154 (82.4%) | 39 (84.8%) | 0.863^1^ |
| ^1^Fisher's exact test  Abbreviations: ABVD, Epirubicin, Bleomycin, Vinorelbine, and Dacarbazine; A-AVD, Brentuximab vedotin, Epirubicin, Vinorelbine, and Dacarbazine; CR = Complete Response; CMR = Complete Metabolic Response; PR = Partial Response; PMR = Partial Metabolic Response; SD = Stable Disease; PD = Progressive Disease; ORR = Objective Response Rate.  Note. 10 patients have missing end evaluations. | | | | |

Supplementary Table S3. Evaluation of interim and end of treatment in patients with Ann Arbor I-II

| Characteristic | Overall  N = 109 | ABVD  N = 94 | A-AVD  N = 15 | p-value |
| --- | --- | --- | --- | --- |
| First (interim) evaluation, n (%) |  |  |  | 0.802^1^ |
| CR | 91 (83.5%) | 77 (81.9%) | 14 (93.3%) |  |
| PR | 15 (13.8%) | 14 (14.9%) | 1 (6.7%) |  |
| PD | 3 (2.8%) | 3 (3.2%) | 0 (0.0%) |  |
| ORR | 106 (97.2%) | 91 (96.8%) | 15 (100.0%) | >0.999^1^ |
| Deauville Score, n (%) |  |  |  | 0.530^1^ |
| 1 | 29 (26.6%) | 25 (26.6%) | 4 (26.7%) |  |
| 2 | 43 (39.4%) | 38 (40.4%) | 5 (33.3%) |  |
| 3 | 19 (17.4%) | 14 (14.9%) | 5 (33.3%) |  |
| 4 | 15 (13.8%) | 14 (14.9%) | 1 (6.7%) |  |
| 5 | 3 (2.8%) | 3 (3.2%) | 0 (0.0%) |  |
| End evaluation, n (%) |  |  |  | 0.691^1^ |
| CR/CMR | 85 (79.4%) | 72 (77.4%) | 13 (92.9%) |  |
| PR/PMR | 4 (3.7%) | 4 (4.3%) | 0 (0.0%) |  |
| PD | 18 (16.8%) | 17 (18.3%) | 1 (7.1%) |  |
| ORR | 89 (83.2%) | 76 (81.7%) | 13 (92.9%) | 0.456^1^ |
| ^1^Fisher's exact test | | | | |

Abbreviations: ABVD, Epirubicin, Bleomycin, Vinorelbine, and Dacarbazine; A-AVD, Brentuximab vedotin, Epirubicin, Vinorelbine, and Dacarbazine; CR = Complete Response; CMR = Complete Metabolic Response; PR = Partial Response; PMR = Partial Metabolic Response; PD = Progressive Disease; ORR = Objective Response Rate.

Note. 2 patients have missing end evaluations.

Supplementary Table S4. Evaluation of interim and end of treatment in patients with Ann Arbor III-IV

| Characteristic | Overall  N = 134 | ABVD  N = 98 | A-AVD  N = 36 | p-value |
| --- | --- | --- | --- | --- |
| First (interim) evaluation, n (%) |  |  |  | 0.536^1^ |
| CR | 79 (59.0%) | 56 (57.1%) | 23 (63.9%) |  |
| PR | 45 (33.6%) | 33 (33.7%) | 12 (33.3%) |  |
| PD | 10 (7.5%) | 9 (9.2%) | 1 (2.8%) |  |
| ORR | 124 (92.5%) | 89 (90.8%) | 35 (97.2%) | 0.287^1^ |
| Deauville Score, n (%) |  |  |  | 0.334^1^ |
| 1 | 22 (16.9%) | 14 (14.9%) | 8 (22.2%) |  |
| 2 | 35 (26.9%) | 29 (30.9%) | 6 (16.7%) |  |
| 3 | 32 (24.6%) | 24 (25.5%) | 8 (22.2%) |  |
| 4 | 32 (24.6%) | 20 (21.3%) | 12 (33.3%) |  |
| 5 | 9 (6.9%) | 7 (7.4%) | 2 (5.6%) |  |
| End evaluation, n (%) |  |  |  | 0.780^1^ |
| CR/CMR | 86 (68.3%) | 63 (67.0%) | 23 (71.9%) |  |
| PR/PMR | 18 (14.3%) | 15 (16.0%) | 3 (9.4%) |  |
| SD | 1 (0.8%) | 1 (1.1%) | 0 (0.0%) |  |
| PD | 21 (16.7%) | 15 (16.0%) | 6 (18.8%) |  |
| ORR | 104 (82.5%) | 78 (83.0%) | 26 (81.3%) | 0.840^1^ |
| ^1^Fisher's exact test  Abbreviations: ABVD, Epirubicin, Bleomycin, Vinorelbine, and Dacarbazine; A-AVD, Brentuximab vedotin, Epirubicin, Vinorelbine, and Dacarbazine; CR = Complete Response; CMR = Complete Metabolic Response; PR = Partial Response; PMR = Partial Metabolic Response; SD = Stable Disease; PD = Progressive Disease; ORR = Objective Response Rate.  Note. 8 patients have missing end evaluations. | | | | |

Supplementary Table S5. Univariate Analysis of Progression-Free Survival

| Characteristic | N | Event N | HR | 95% CI | p-value |
| --- | --- | --- | --- | --- | --- |
| Gender |  |  |  |  |  |
| Male | 127 | 47 | — | — |  |
| Female | 116 | 26 | 0.57 | 0.35, 0.92 | 0.023 |
| Age |  |  |  |  |  |
| ˂ 60 | 212 | 62 | — | — |  |
| ≥ 60 | 31 | 11 | 1.34 | 0.70, 2.54 | 0.375 |
| Pathology |  |  |  |  |  |
| Mixed Cellularity | 48 | 21 | — | — |  |
| Nodular Sclerosis | 130 | 35 | 0.56 | 0.33, 0.97 | 0.037 |
| Lymphocyte-Rich | 34 | 8 | 0.46 | 0.20, 1.05 | 0.065 |
| Lymphocyte-Depleted | 2 | 0 | 0.00 | 0.00, Inf | 0.995 |
| Unclassifiable | 29 | 9 | 0.69 | 0.31, 1.51 | 0.353 |
| Ann Arbor |  |  |  |  |  |
| I | 8 | 2 | — | — |  |
| II | 101 | 21 | 0.47 | 0.11, 2.02 | 0.311 |
| III | 42 | 13 | 0.75 | 0.17, 3.33 | 0.703 |
| IV | 92 | 37 | 0.97 | 0.23, 4.06 | 0.972 |
| B symptoms |  |  |  |  |  |
| A | 128 | 32 | — | — |  |
| B | 115 | 41 | 1.49 | 0.94, 2.36 | 0.094 |
| Bulky Disease |  |  |  |  |  |
| No | 154 | 46 | — | — |  |
| Yes | 89 | 27 | 1.16 | 0.72, 1.88 | 0.541 |
| Lung Involvement |  |  |  |  |  |
| No | 197 | 55 | — | — |  |
| Yes | 46 | 18 | 1.46 | 0.85, 2.48 | 0.169 |
| Bone Involvement |  |  |  |  |  |
| No | 183 | 49 | — | — |  |
| Yes | 60 | 24 | 1.54 | 0.95, 2.51 | 0.083 |
| Spleen Involvement |  |  |  |  |  |
| No | 198 | 57 | — | — |  |
| Yes | 45 | 16 | 1.19 | 0.68, 2.08 | 0.534 |
| Liver Involvement |  |  |  |  |  |
| No | 224 | 62 | — | — |  |
| Yes | 19 | 11 | 2.28 | 1.20, 4.33 | 0.012 |
| Treatment Regimen |  |  |  |  |  |
| ABVD | 192 | 62 | — | — |  |
| A-AVD | 51 | 11 | 0.80 | 0.42, 1.52 | 0.490 |
| Abbreviations: ABVD, Epirubicin, Bleomycin, Vinorelbine, and Dacarbazine; A-AVD, Brentuximab vedotin, Epirubicin, Vinorelbine, and Dacarbazine; PFS = Progression-Free Survival; HR = Hazard Ratio; CI = Confidence Interval; Ref = Reference group; Inf = Infinity (indicates the upper bound of the confidence interval could not be estimated).  Notes:  a) Hazard ratios and 95% confidence intervals were estimated using univariate Cox proportional-hazards regression models.  b) "NA" or dashes indicate that the HR could not be reliably estimated due to zero events or an extremely small sample size in the respective subgroup. | | | | | |

Supplementary Table S6. Adverse events in ABVD, A-AVD and all.

| Characteristic | Overall N = 243 | ABVD N = 192 | A-AVD N = 51 | p-value |
| --- | --- | --- | --- | --- |
| Neutropenia, n (%) | 73 (30.0%) | 55 (28.6%) | 18 (35.3%) | 0.355^1^ |
| Grade 1-2 | 29 (11.9%) | 21 (10.9%) | 8 (15.7%) | 0.347^1^ |
| Grade 3-4 | 44 (18.1%) | 34 (17.7%) | 10 (19.6%) | 0.751^1^ |
| Anemia, n (%) | 42 (17.3%) | 31 (16.1%) | 11 (21.6%) | 0.362^1^ |
| Grade 1-2 | 25 (10.3%) | 20 (10.4%) | 5 (9.8%) | 0.895^1^ |
| Grade 3-4 | 17 (7.0%) | 11 (5.7%) | 6 (11.8%) | 0.171^2^ |
| Thrombocytopenia, n (%) | 40 (16.5%) | 28 (14.6%) | 12 (23.5%) | 0.128^1^ |
| Grade 1-2 | 27 (11.1%) | 21 (10.9%) | 6 (11.8%) | 0.858^1^ |
| Grade 3-4 | 13 (5.3%) | 7 (3.6%) | 6 (11.8%) | 0.041^2^ |
| Fever, n (%) | 23 (9.5%) | 17 (8.9%) | 6 (11.8%) | 0.528^2^ |
| Grade 1-2 | 13 (5.3%) | 11 (5.7%) | 2 (3.9%) | 0.741^2^ |
| Grade 3-4 | 10 (4.1%) | 6 (3.1%) | 4 (7.8%) | 0.126^2^ |
| Infection, n (%) | 43 (17.7%) | 30 (15.6%) | 13 (25.5%) | 0.103^1^ |
| Grade 1-2 | 18 (7.4%) | 10 (5.2%) | 8 (15.7%) | 0.015^2^ |
| Grade 3-4 | 25 (10.3%) | 20 (10.4%) | 5 (9.8%) | 0.895^1^ |
| Nausea, n (%) | 39 (16.0%) | 23 (12.0%) | 16 (31.4%) | 0.001^1^ |
| Grade 1-2 | 35 (14.4%) | 21 (10.9%) | 14 (27.5%) | 0.004^1^ |
| Grade 3-4 | 4 (1.6%) | 2 (1.0%) | 2 (3.9%) | 0.185^2^ |
| Hepatic Toxicity, n (%) | 34 (14.0%) | 25 (13.0%) | 9 (17.6%) | 0.392^1^ |
| Grade 1-2 | 24 (9.9%) | 17 (8.9%) | 7 (13.7%) | 0.301^1^ |
| Grade 3-4 | 10 (4.1%) | 8 (4.2%) | 2 (3.9%) | >0.999^2^ |
| Peripheral Neuropathy, n (%) | 34 (14.0%) | 10 (5.2%) | 24 (47.1%) | <0.001^1^ |
| Grade 1-2 | 29 (11.9%) | 10 (5.2%) | 19 (37.3%) | <0.001^1^ |
| Grade 3-4 | 5 (2.1%) | 0 (0.0%) | 5 (9.8%) | <0.001^2^ |
| Pulmonary Toxicity, n (%) | 19 (7.8%) | 18 (9.4%) | 1 (2.0%) | 0.125^2^ |
| Grade 1-2 | 9 (3.7%) | 8 (4.2%) | 1 (2.0%) | 0.692^2^ |
| Grade 3-4 | 10 (4.1%) | 10 (5.2%) | 0 (0.0%) | 0.111^2^ |
| Rash, n (%) | 23 (9.5%) | 12 (6.2%) | 11 (21.6%) | 0.001^2^ |
| Grade 1-2 | 17 (7.0%) | 9 (4.7%) | 8 (15.7%) | 0.015^2^ |
| Grade 3-4 | 6 (2.5%) | 3 (1.6%) | 3 (5.9%) | 0.091^2^ |
| All Adverse Events, n (%) | 139 (57.2%) | 108 (56.2%) | 31 (60.8%) | 0.551^1^ |
| Grade 1-2 | 80 (32.9%) | 66 (34.4%) | 14 (27.5%) | 0.354^1^ |
| Grade 3-4 | 59 (24.3%) | 42 (21.9%) | 17 (33.3%) | 0.093^1^ |

^1^Pearson's Chi-squared test

^2^Fisher's exact test

Abbreviations: ABVD, Epirubicin, Bleomycin, Vinorelbine, and Dacarbazine; A-AVD, Brentuximab vedotin, Epirubicin, Vinorelbine, and Dacarbazine; CTCAE = Common Terminology Criteria for Adverse Events.

Notes: a) Adverse events were graded according to the National Cancer Institute CTCAE (Version 5.0).

b) P-values represent the comparison of Grade 3-4 adverse events between the two treatment arms.
